# High Ki-67 Expression Predicting a Risk Factor for the Progression of Disease within 24 Months and Microenvironment in Follicular Lymphoma

**DOI:** 10.3390/ijms252011057

**Published:** 2024-10-15

**Authors:** Hinako Narita, Kai Kuroiwa, Yukiko Kawaguchi, So Murai, Yosuke Sasaki, Mayumi Homma, Natsuki Kawamata, Hidenori Hayashi, Kazuki Nagao, Reiko Okamura, Yuka Uesugi, Yohei Sasaki, Shotaro Shimada, Megumi Watanuki, Nana Arai, Kouji Yanagisawa, Eisuke Shiozawa, Toshiko Yamochi, Norimichi Hattori

**Affiliations:** 1 Division of Hematology, Department of Medicine, Showa University School of Medicine, Tokyo 142-8666, Japan; gm23-h032@med.showa-u.ac.jp (H.N.); gm21-k021@med.showa-u.ac.jp (K.K.); kawaguchi.y@med.showa-u.ac.jp (Y.K.); natsuki.k@med.showa-u.ac.jp (N.K.); l6m0090e@med.showa-u.ac.jp (H.H.); k.nagao0828@med.showa-u.ac.jp (K.N.); rokamura@med.showa-u.ac.jp (R.O.); y-uesugi@med.showa-u.ac.jp (Y.U.); ysasaki@med.showa-u.ac.jp (Y.S.); s.shima09m@med.showa-u.ac.jp (S.S.); m.watanuki11@med.showa-u.ac.jp (M.W.); nana.arai@med.showa-u.ac.jp (N.A.); yanagik@med.showa-u.ac.jp (K.Y.); 2 Department of Pathology and Laboratory Medicine, Showa University School of Medicine, Tokyo 142-8666, Japan; smurai1015@med.showa-u.ac.jp (S.M.); sasaki0224@med.showa-u.ac.jp (Y.S.); mayumi-h@med.showa-u.ac.jp (M.H.); shiozawa@med.showa-u.ac.jp (E.S.); onizuka@med.showa-u.ac.jp (T.Y.)

**Keywords:** Ki-67, POD24, follicular lymphoma, tumor microenvironment, macrophage

## Abstract

Most follicular lymphomas (FLs) demonstrate an indolent clinical course with favorable outcomes; however, a fraction of patients experiences progression of disease within 24 months (POD24) and has adverse outcomes. This study aimed to determine the predictive risk factors for POD24 in patients with FL, and the characteristics of the microenvironment in FL with POD24. By multivariate analysis, we revealed that increased Ki-67 expression was associated with POD24 events in patients with FL (hazard ratio [HR]: 6.29, 95% confidence interval [CI]: 1.96–20.22, *p* = 0.0020). Additionally, patients with FL with POD24 demonstrated immune cell reduction by immunohistochemistry analysis. Our results help better understand the therapeutic strategies for FL with POD24.

## 1. Introduction

Follicular lymphoma (FL), the most prevalent type of indolent non-Hodgkin lymphoma, usually demonstrates indolent clinical behavior. While most patients undergo an indolent clinical course characterized by relapsing and remitting patterns, early relapse in FL, defined as disease progression or recurrence within 24 months of first-line treatment (POD24), affects around 20% of patients and is linked to a poor prognosis [1,2,3]. Therefore, predicting POD24 before first-line treatment could benefit patients. Several clinical prognostic tools are available to predict outcomes, including the Follicular Lymphoma International Prognosis Index (FLIPI) [4], FLIPI-2 [5], PRIMA-prognostic index (PRIMA-PI) [6], and Follicular Lymphoma Evaluation Index (FLEX) [7]. These models were developed based on patients receiving alkylating agents but did not incorporate bendamustine, which is frequently used for patients with FL. The FLEX model is constrained by the routine lack of several calculated parameters despite the inclusion of patients treated with obinutuzumab and bendamustine (GB) [7,8]. Moreover, several genetic models that include the status of several gene mutations or gene-expression profiling have been proposed to develop clinical risk models, such as m7-FLIPI [9], POD24-PI [10], the 23-gene signature model [11], and Bio-clinical FLIPI (Bio-FLIPI) [12]. However, these models are not standardized and remain part of a research approach. In a recent study comparing the FLIPI, FLIPI2, PRIMA-PI, and m7-FLIPI models, it was found that FLIPI remained a PI with higher discriminatory power for survival in patients with advanced FL treated with immunochemotherapy, including bendamustine plus rituximab (BR) [13]. Overall, optimal tools to estimate prognostic factors, in particular, POD24 for patients with FL, remain under development.

The tumor microenvironment of FL includes a variable number of immune cells that contribute to immunosurveillance and immune evasion. However, studies have reported discrepant results regarding the infiltration of specific immune cells, with some showing a correlation with a better prognosis while others indicate a poorer prognosis [14,15,16,17,18]. The present study investigated the predictive risk factors for POD24 and explored the characteristics of patients with FL with POD24, particularly focusing on the microenvironment.

## 2. Results

### 2.1. Patient Characteristics

Table 1 summarizes the characteristics of 101 patients. Our patients had a median age of 65 years at diagnosis. Additionally, 80 patients (79%) received R + chemotherapy; including rituximab plus cyclophosphamide, doxorubicin, vincristine, and prednisolone (R-CHOP) (*n* = 29); R plus cyclophosphamide, pirarubicin, vincristine, and prednisolone (R-THP-COP) (*n* = 29); and R plus cyclophosphamide, vincristine, and prednisone (R-CVP) (*n* = 6), and BR (*n* = 16). Moreover, nine patients (8%) received R monotherapy. Other therapies included GB (*n* = 9), fludarabine (*n* = 2), and CVP (*n* = 1).

**Figure 1:**
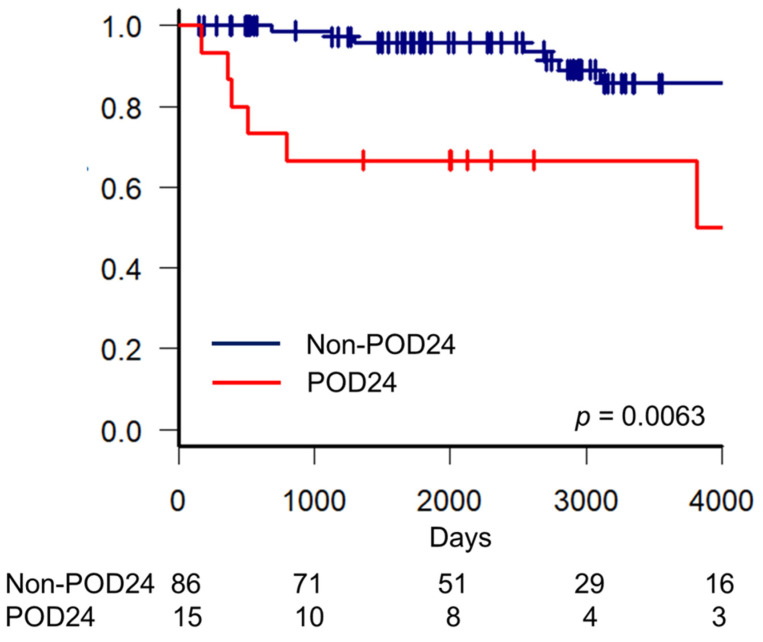
Overall survival in patients with FL with and without POD24.

The median follow-up of this study population was 2483 days (range: 149–7224), with 85 (84.2%) patients who were alive and 16 (15.8%) who died because of relapse or disease progression (*n* = 13), infections (*n* = 2), and aortic dissection (*n* = 1). Additionally, 40 (39.6%) patients had a relapse, of which 10 (10/40, 25.0%) had progression to DLBCL. Moreover, of 40 patients, 15 (15/40, 37.5%) were classified as POD24. Of them, four (4/15, 26.7%) had progression to DLBCL. In 15 patients with POD24, no patient (0/25, 0%) received RB or GB, and 12 patients (12/65, 18.5%) received R-CHOP, R-THP-COP, R-CVP, or CVP as first-line treatment. Of 40 relapsed patients, 2 (2/40, 5.0%) received autologous stem cell transplantation (ASCT), and 1 (1/40, 2.5%) received allogeneic stem cell transplantation as salvage therapy after relapse. Of the 15 patients with POD24, 1 (1/15, 6.7%) received ASCT. Furthermore, 5-year OS rates in patients with POD24 and without POD24 were 66.7% (95% confidence interval [CI]: 37.5–84.6%) and 95.8% (95% CI: 87.4–98.6%), respectively. Patients with POD24 had a significant adverse effect on OS compared with those without POD24 (*p* = 0.0063) (Figure 1).

### 2.2. Comparison of Characteristics between Patients with FL with and without POD24

Table 2 shows the characteristics of the subgroups with POD24 and without POD24. The POD24 group compared with the non-POD24 group was enriched for higher Ki-67 expression (73% vs. 22%, *p* = 2.1 × 10^−4^). No differences in other parameters, including the FLIPI score, LDH, sIL-2R, β2-MG level, or high tumor burden, were found between the two groups.

Subsequently, univariate and multivariate analyses were conducted to clarify the predictive risk factors for POD24 (Table 3). The univariate analysis showed that elevated LDH level (hazard ratio [HR]: 2.78, 95% CI: 1.01–7.67, *p* = 0.049) and high Ki-67 expression (HR: 7.17, 95% CI: 2.28–22.55, *p* = 7.4 × 10^−4^) were related to the occurrence of POD24. In the multivariate analysis, only high Ki-67 expression was associated with POD24 occurrence (HR: 6.29, 95% CI: 1.96–20.22, *p* = 0.0020).

### 2.3. Association between the Microenvironment and POD24

**Figure 2:**
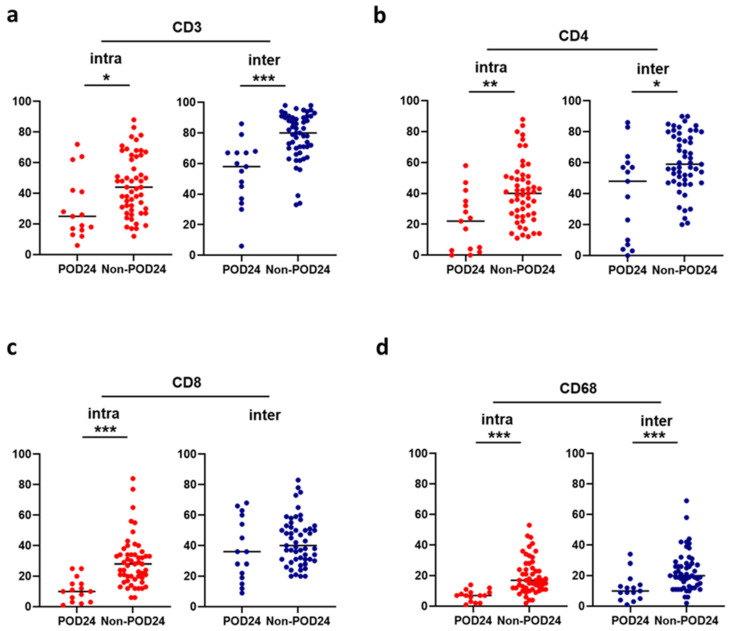
Comparison of immune cells between patients with FL with and without POD24 according to separately counted intrafollicular and interfollicular areas. (**a**) CD3, (**b**) CD4, (**c**) CD8, and (**d**) CD68. * *p* < 0.05, ** *p* < 0.01, and *** *p* < 0.001.

We compared the proportions of microenvironment components in two distinct areas, which were categorized into intrafollicular and interfollicular between patients with and without POD24, to investigate potential associations between the microenvironment and the occurrence of POD24 (Figure 2). Compared with patients with POD24, patients without POD24 demonstrated a higher proportion of CD3-positive cells in intrafollicular (mean: 45% vs. 31%; *p* = 0.024) and interfollicular (mean: 78% vs. 54%; *p* = 5.1 × 10^−4^), CD4-positive cells in intrafollicular (mean: 41% vs. 21%; *p* = 0.0018) and interfollicular (mean: 60% vs. 40%; *p* = 0.019), CD8-positive cells in intrafollicular (mean: 29% vs. 11%; *p* = 1.7 × 10^−7^), and CD68-positive cells in intrafollicular (mean: 20% vs. 7%; *p* = 3.2 × 10^−9^) and interfollicular (mean: 23% vs. 12%; *p* = 7.9 × 10^−4^). In addition, Ki-67 expression by immunohistochemistry analysis, which was separated from Ki-67 expression in clinical data, was evaluated. Compared with patients without POD24, patients with POD24 demonstrated a higher proportion of Ki-67-positive cells in intrafollicular (mean: 52% vs. 31%; *p* = 0.0092). There was no difference in interfollicular Ki-67 expression (mean: 23% vs. 22%; *p* = 0.92) (Figure S1).

### 2.4. Relationship between Microenvironment Components and Clinical Outcomes

Next, we investigated the relationship between the microenvironment components in two distinct areas and clinical outcomes using multivariate analysis (Table 4). Reduced CD3-positive cells in interfollicular (HR: 0.95, 95% CI: 0.92–0.99, *p* = 0.022) and CD68-positive cells in intrafollicular (HR: 0.85, 95% CI: 0.72–0.99, *p* = 0.045) were significantly associated with higher risk of POD24 development. Reduced CD3-positive cells in interfollicular (HR: 0.95, 95% CI: 0.93–0.98, *p* = 0.0012) and CD8-positive cells in intrafollicular (HR: 0.92, 95% CI: 0.86–0.97, *p* = 0.0038) were significantly associated with poorer progression-free survival (PFS). There was statistically no association between microenvironment components and overall survival (OS).

## 3. Discussion

We revealed high Ki-67 expression as a predictive risk factor for POD24 in patients with FL. Several studies revealed that patients with FL exhibiting high Ki-67 expression (≥30%) demonstrated inferior outcomes [19,20,21]. However, only a few studies reported the association between high Ki-67 expression and POD24 [21]. Consistent with a previous report [21], we revealed high Ki-67 expression as a simple and efficient predictive factor for POD24. Additionally, we observed higher Ki-67 expression in the intrafollicular area in patients with POD24 compared with those without POD24 [20], while no difference was noted in interfollicular Ki-67 expression. Nasir et al. [22] revealed that high Ki-67 expression in the interfollicular area was associated with inferior outcomes, whereas Klapper et al. [23] demonstrated no correlation between interfollicular Ki-67 and clinical outcomes, consistent with our findings. Immune cells such as T cells do not only exist in interfollicular, but they also exist in intrafollicular. Meanwhile, tumor B cells can spread to the interfollicular area in some FL patients. Thus, it can be unclear whether Ki67 was stained with tumor B cells or activated T cells. Future studies are warranted to identify whether cells stained with Ki-67 spread tumor B cells or activate immune cells because various cells exist in interfollicular. Our study had some limitations, including our cohort being from a single center, the retrospective study design, patients receiving different first-line treatments, and molecular data, such as gene mutations, were not assessed, in addition to a lack of some clinical data including β2-MG. In fact, an elevated baseline level of β2-MG was included in FLIPI2 or PRIMA-PI as the prognostic marker [5,6]. Thus, further validation is required.

Our findings for OS at 5 years of 66.7% and 95.8% in patients with POD24 and without POD24, respectively, were similar to those of FLASH data analysis (71.2% and 93.6%, respectively) [2]. However, compared with FLASH data, our cohort included much older patients with higher rates of patients receiving bendamustine and immunotherapies such as rituximab and obinutuzumab. Notably, rates of POD24 occurrence in patients treated with bendamustine-based therapies (0%) were lower than those with alkylator-based regimens (18.5%). This observation was consistent with previous reports [3,24]. Thus, bendamustine-based therapies as first-line treatment may decrease the incidence of POD24; however, the median follow-up in patients with bendamustine-based therapies was shorter than those with alkylator-based regimens in our study (861 days vs. 2948 days). However, previous reports [3,24] showed a high risk of developing transformation to DLBCL in FL with POD24 after frontline bendamustine-based therapies. In particular, early progression after BR therapy may have a high risk of transformation to DLBCL. Therefore, the treatment strategy for improving clinical outcomes in FL patients still remains.

The significant finding in the present study is that patients with FL with POD24 demonstrated reduced numbers of immune cells compared with those without POD24. A previous report on the more frequent T cell infiltration in FL with spontaneous remission, lower numbers of immune cells in high-risk FL, the role of memory and naive T cells for maintenance of antitumor immunity, and low immune infiltration related to experience POD24 events support our data [14,16,17,25,26]. Additionally, corroborating our observations, a recent study with immune infiltration by intratumoral T cells quantified ^18^F-fluorodeoxyglucose–PET revealed that high total metabolic tumor volume reflected an inverse association with the numbers of intratumoral CD4- and CD8-positive T cells and a correlation with increased tumor B cell infiltration and high Ki-67 expression [27]. Similarly, Bio-FLIPI determined that only a lack of intrafollicular CD4 expression predicts early failure [12]. Moreover, a previous single-cell RNA sequencing study revealed the existence of cytotoxic CD4 T cells in the FL microenvironment and a T cell-depleted microenvironment with an inferior clinical outcome [17]. Conversely, CD4-positive T follicular helper (Tfh) cells are enriched in FL and generally support tumor growth in FL [28]. However, a recent study on the FL organoid model revealed a significant positive correlation between Tfh activation and tumor-killing after bispecific antibody treatment [29]. Thus, Tfh cell dysfunction, including immune activation, suppression, and exhaustion may be more complicated in FL. The subsets of CD4-positive cells were unspecified in our study, and there was no association between the proportion of simple CD4-positive cells and clinical outcomes; thus, further studies are warranted.

We showed that patients with decreased CD8-positive cells had inferior PFS and a tendency for POD24 development. Similar to our results, Rai et al. [30] demonstrated that CD8-positive T cell markers (*CD8A*, *CD8B*, *FLT3LG*, *GZMM*, and *GZMK*) were downregulated in FL patients with POD24, while NK cell markers and immune checkpoint markers were almost equivalently expressed in those with or without POD24. Liu et al. [31] showed that decreased CD8-positive T cells with upregulated LAG-3 expression around FL-cells were observed in intrafollicular during POD24. Also, Alvaro et al. [14] showed that FL patients with higher infiltration of CD8-positive T cells had a favorable prognosis. These findings suggest immune cells such as CD8-positive T cells have a potential impact on survival and risk of POD24 in FL patients. In fact, the combination of lenalidomide plus rituximab [32] or obinutuzumab [33] in patients with untreated FL patients has produced promising results. The RELEVANCE trial (phase II study) on the combination of lenalidomide plus rituximab in untreated FL patients showed that the 2-year PFS was 86% and the 5-year OS was 100% [32]. A LYSA study (phase II study) on the combination of obinutuzumab and lenalidomide in untreated FL patients reported that 3-year PFS was 82% and OS was 94% [33]. Lenalidomide is an immunomodulator, and T cells and NK cells stimulated by lenalidomide may enhance antibody-dependent cellular cytotoxicity and cytotoxic T cell activity. In addition, lenalidomide has been shown to produce synergistic effects in combination with rituximab, dexamethasone, bortezomib, and B cell receptor signaling inhibitors [34]. Therefore, the evaluation of immune cells in FL patients may be important for exploring effective treatment options.

In our study, patients with reduced CD3-positive cells in interfollicular had poorer PFS and a higher risk for POD24 occurrence. Xerri et al. [35] reported that low CD3 and low PD1 counts in malignant follicles containing intrafollicular and interfollicular were associated with inferior PFS using FL samples from rituximab-treated patients enrolled in the randomized PRIMA trial. Additionally, RNAseq analysis showed a correlation between low CD3 and CD8 expression and poorer PFS. According to separately counted intrafollicular and interfollicular in our study, we found that only CD3-positive cells in interfollicular had a prognostic value. CD3 is a pan T cell marker covering a broad range of potentially complementary and antagonistic T cell subsets (e.g., regulatory T cells). Nevertheless, we suggest that CD3-positive cells in interfollicular can be a helpful tool for predicting prognosis.

The impact of macrophages that contributed to clinical outcomes is different in patients with FL treated with and without rituximab [14,15,16,36]. These observations indicated that the clinical impact of macrophages in patients with FL may depend on the administered agents, such as the anti-CD20 antibody, accentuating the requirement for a better understanding of macrophages that mediate antitumoral activity in FL. Tobin et al. [16] revealed that low expression of immune infiltrating cells, including macrophage markers, was correlated with a higher risk of POD24 events, consistent with our results, including most patients treated with immunochemotherapy. Additionally, a current study on multi-omic profiling demonstrated that the microenvironment in patients with high-risk FL changed to a loss of follicular dendritic cell meshwork, a reduction in macrophages, and the expansion of stromal cells [37]. Recently, CD47 blockade (Hu5F9-G4 or magrolimab), which is a macrophage checkpoint inhibitor that induces macrophage-mediated tumor killing, combined with rituximab [38] showed potential efficacy in patients with relapsed or refractory FL. The rates of complete response and partial response were 43% and 29%, respectively. Considering our findings that reduced macrophages were found in patients with POD24, the response to the combination of 5F9 and rituximab may depend on the proportion of macrophages in intrafollicular.

In conclusion, we revealed that increased Ki-67 expression before FL treatment can be a hallmark of POD24 events. Furthermore, patients with FL with POD24 have reduced immune cells in the microenvironment. These results can effectively contribute to future therapeutic strategies for FL.

## 4. Materials and Methods

### 4.1. Patients

This analysis enrolled 101 untreated patients aged 30–86 years who were newly diagnosed with FL at our institution from 2003 to 2021. Following the World Health Organization classification, expert pathologists performed histologic diagnosis. This study excluded patients with histologic grade 3b FL, defined as histologic transformation to diffuse large B cell lymphoma (DLBCL). Moreover, in situ follicular B cell neoplasm, pediatric FL, or duodenal-type FL were excluded [39]. Patients were staged following the Ann Arbor classification and Eastern Cooperative Oncology Group performance status (PS). High tumor burden, according to GELF criteria, was defined as at least one of the parameters including B symptoms, involvement of ≥3 nodal sites (each > 3 cm), any tumor mass over 7 cm, splenomegaly, organ compression by tumor, pleural/peritoneal effusion, leukemic phase, or cytopenias [40]. All measurable lesions were evaluated by computed tomography (CT) or positron emission tomography (PET)/CT, physical examination, and bone marrow biopsy. Patient response after induction chemotherapy was classified according to the International Workshop criteria [41]. Clinical data, including age, sex, histological grade, stage, PS, FLIPI, hemoglobin, lactate dehydrogenase (LDH), soluble interleukin-2 receptor (sIL-2R), and β2-microglobulin (β2-MG), were collected at diagnosis. Furthermore, Ki-67 was included in the clinical data, which is routinely used for pathological diagnosis. High Ki-67 expression was defined as ≥30% based on previously described [19,42]. This study was conducted in accordance with the Declaration of Helsinki, and the protocol was approved by the Ethics Committee of Showa University, Tokyo, Japan, (No. 22-251-A) on 30 January 2023.

### 4.2. Histology and Immunohistochemistrical Analysis

**Figure 3:**
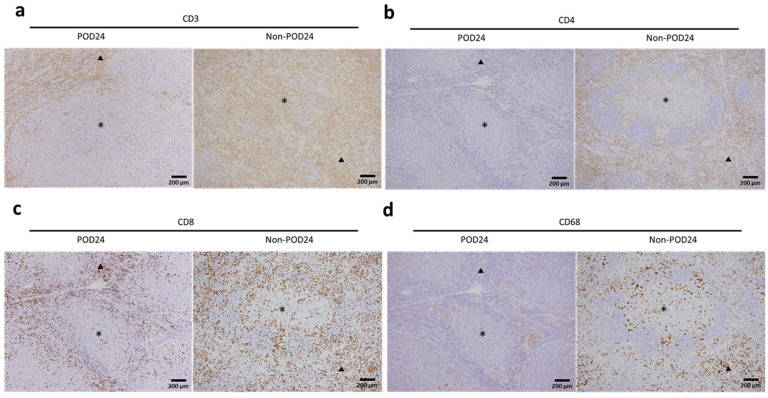
Immunohistochemical staining (magnification, 100×) of the intrafollicular area (black asterisks) and interfollicular area (black triangles) in samples of patients with FL and POD24 (66 years, male) and non-POD24 (63 years, female). Staining for (**a**) CD3, (**b**) CD4, (**c**) CD8, and (**d**) CD68.

All samples were collected at the time of initial diagnosis. Hematoxylin and eosin staining of 3 µm sections was used to evaluate the histopathological findings of tumors. Formalin-fixed paraffin-embedded sections stained with the following antibodies were used for the immunohistochemistry analysis: CD3 (clone 565, 1:200; Novocastra, Newcastle, U.K.), CD4 (clone 1F6, 1:40; Novocastra, Newcastle, U.K.), CD8 (C8/144B, 1:100; Dako, Glostrup, Denmark), CD68 (KP1, 1:200; Dako, Glostrup, Denmark), and Ki-67 (clone MIB-1, 1:200; Dako, Glostrup, Denmark). An automated immunostainer (Histostainer 36 A, Nichirei Biosciences Inc., Tokyo, Japan) was used for immunostaining following the manufacturer’s protocol. Samples for this analysis were available in 15 patients with POD24 and 53 patients without POD24. Initially, a low-power magnification (magnification 100×) was used to determine the number of CD3-, CD4-, CD8-, and CD68-positive cells. The positive cells were evaluated in two distinct areas, including intrafollicular- and interfollicular-positive cell counts, considering the various numbers of these cells inside and between the neoplastic follicles. Both intrafollicular- and interfollicular-positive cells were counted separately on high power (magnification 400×) from three different hot spots per case (Figure 3). Two different pathologists, blinded to the patient background, independently performed the counts. The statistical analysis incorporated the mean percentages of cells expressing each marker divided by all nucleated cells in three distinct hot spots [18,43,44,45].

### 4.3. Definitions and Statistics

POD24 was disease progression or relapse within 24 months from treatment initiation (modified definition) [10], PFS was the time from the date of diagnosis to relapse, progression, or death from any cause, and OS was the time between the date of diagnosis and the patient’s death from any cause or last follow-up. Progression in lymphoma is defined as ≥50% in the area from the nadir, which indicates that a 22.5% increase in each of the maximal and perpendicular axes is needed for a 50% increase in area.

Laboratory parameters were dichotomized using the lower and upper limits of normal established in our hospital. To compare intergroup differences, the chi-square test or Fisher’s exact test was used for categorical variables and the Kruskal–Wallis test was used for continuous variables. The log-rank test for OS was used for univariate analyses. Univariate and multivariate Cox regression analyses were used to evaluate the predictive risk factors of POD24. Statistical significance was considered at a *p*-value of <0.05. EZR [46] and GraphPad Prism 8 (GraphPad Software Inc., San Diego, CA, USA) were used for statistical analysis.

## Figures and Tables

**Table 1:** Patient characteristics.

Parameter		*n* (%)
Number of patients		101 (100)
Age		
	<60 years	28 (28)
	≥60 years	73 (72)
Sex		
	Female	50 (50)
	Male	51 (50)
ECOG PS		
	0–1	94 (93)
	2–4	7 (7)
Ann Arbor		
	Stage I/II	22 (22)
	Stage III/IV	79 (78)
Histological findings		
	Grade 1–2	80 (79)
	Grade 3a	21 (21)
FLIPI		
	Low risk	26 (26)
	Intermediate risk	34 (34)
	High risk	41 (41)
Hb		
	<LLN (g/dL)	21 (21)
	≥LLN (g/dL)	80 (79)
LDH		
	<ULN (U/L)	69 (68)
	≥ULN (U/L)	32 (32)
sIL-2R		
	<ULN (U/mL)	28 (28)
	≥ULN (U/mL)	72 (71)
	Data missing	1 (1)
β2-MG		
	<ULN (mg/L)	37 (37)
	≥ULN (mg/L)	25 (25)
	Data missing	39 (39)
Ki-67		
	Low (<30%)	70 (69)
	High (≥30%)	30 (30)
	Data missing	1 (1)
High tumor burden by GELF criteria		39 (39)
POD24		
	POD24	15 (15)
	Non-POD24	86 (85)
First treatment		
	Rituximab + chemotherapy	80 (79)
	Rituximab monotherapy	9 (9)
	Other	12 (12)

Abbreviations: ECOG PS: Eastern Cooperative Oncology Group performance status; FLIPI: Follicular Lymphoma International Prognostic Index; Hb: hemoglobin; LLN: lower limit of normal; sIL-2R: soluble interleukin-2 receptor; ULN: upper limit of normal; β2-MG: beta-2 macroglobulin; GELF: Groupe d’Etude des Lymphomes Folliculaires; POD24: progression of disease within 24 months of front line.

**Table 2:** Patient characteristics based on POD24 status.

Factor		POD24	Non-POD24		*p*-Value
		(*n* = 15), *n* (%)	(*n* = 86), *n* (%)		
Age					
	<60 years	3 (20)	25 (29)		0.55
	≥60 years	12 (80)	61 (71)		
	Median (range), years	65 (48–83)	65 (30–86)		0.53
Sex					
	Female	7 (47)	43 (50)		1.00
	Male	8 (53)	43 (50)		
Performance status (ECOG)					
	0–1	13 (87)	81 (94)		0.28
	2–4	2 (13)	5 (6)		
Ann Arbor					
	Stage I/II	3 (20)	19 (22)		1.00
	Stage III/IV	12 (80)	67 (78)		
Histological findings					
	Grade 1–2	11 (73)	69 (80)		0.51
	Grade 3a	4 (27)	17 (20)		
FLIPI					
	Low risk	2 (13)	24 (28)		0.36
	Intermediate risk	7 (47)	27 (31)		
	High risk	6 (40)	35 (41)		
Hemoglobin					
	<LLN (g/dL)	4 (27)	17 (20)		0.37
	≥LLN (g/dL)	11 (73)	69 (80)		
LDH					
	<ULN (U/L)	7 (47)	62 (72)		0.071
	≥ULN (U/L)	8 (53)	24 (28)		
sIL-2R					
	<ULN (U/mL)	2 (13)	26 (30)	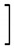	0.22
	≥ULN (U/mL)	13 (87)	59 (69)		
	data missing	0 (0)	1 (1)		
β2-MG					
	<ULN (mg/L)	2 (13)	35 (41)	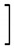	0.052
	≥ULN (mg/L)	6 (40)	19 (22)		
	Data missing	7 (47)	32 (37)		
Ki-67					
	Low (<30%)	4 (27)	66 (77)	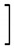	2.1 × 10^−4^
	High (≥30%)	11 (73)	19 (22)		
	Data missing	0 (0)	1 (1)		
High tumor burden by GELF criteria		8 (53)	31 (36)		0.25
First treatment group					
	Rituximab + chemotherapy	11 (73)	69 (80)		0.68
	Rituximab monotherapy	2 (13)	7 (8)		
	Other	2 (13)	10 (12)		

Abbreviations: POD24: progression of disease within 24 months of front line; ECOG Eastern Cooperative Oncology Group; FLIPI: Follicular Lymphoma International Prognostic Index; LLN: lower limit of normal; sIL-2R: soluble interleukin-2 receptor; ULN: upper limit of normal; β2-MG: beta-2 macroglobulin; GELF: Groupe d’Etude des Lymphomes Folliculaires.

**Table 3:** Univariate and multivariate analyses of predictive risk factors for POD24.

		*n*	POD24 Univariate			POD24 Multivariate		
			HR	95% CI	*p*	HR	95% CI	*p*
Age								
	<60 years	28	1					
	≥60 years	73	1.67	0.47–5.93	0.43			
Sex								
	Female	50	1					
	Male	51	1.06	0.38–2.91	0.92			
ECOG PS								
	0–1	94	1					
	2–4	7	2.31	0.52–10.24	0.27			
Ann Arbor								
	Stage I/II	22	1					
	Stage III/IV	79	1.26	0.36–4.47	0.72			
Histological findings								
	Grade 1–2	80	1					
	Grade 3a	21	1.42	0.45–4.47	0.55			
FLIPI								
	Low/Int	60	1					
	High	41	1.14	0.41–3.21	0.80			
Hb								
	<LLN (g/dL)	21	1					
	≥LLN (g/dL)	80	0.62	0.20–1.96	0.42			
LDH								
	<ULN (U/L)	69	1			1		
	≥ULN (U/L)	32	2.78	1.01–7.67	0.049	1.87	0.66–5.27	0.24
sIL-2R								
	<ULN (U/mL)	28	1					
	≥ULN (U/mL)	72	2.84	0.64–12.57	0.17			
Ki-67								
	Low (<30%)	70	1			1		
	High (≥30%)	30	7.17	2.28–22.55	7.4 × 10^−4^	6.29	1.96–20.22	0.0020
High tumor burden								
	No	62	1					
	Yes	39	2.00	0.72–5.51	0.18			

Abbreviations: POD24: progression of disease within 24 months of front line; ECOG PS: Eastern Cooperative Oncology Group performance status; FLIPI: Follicular Lymphoma International Prognostic Index; LLN: lower limit of normal; sIL-2R: soluble interleukin-2 receptor; ULN: upper limit of normal.

**Table 4:** Association between immune cells and clinical outcomes by multivariate analysis.

			POD24			PFS			OS		
			HR	95% CI	*p*	HR	95% CI	*p*	HR	95% CI	*p*
CD3^+^ cells in intrafollicular			1.01	0.97–1.05	0.59	1.01	0.99–1.04	0.41	1.01	0.96–1.06	0.80
in interfollicular			0.95	0.92–0.99	0.022	0.95	0.93–0.98	0.0012	0.96	0.92–1.01	0.14
CD4^+^ cells in intrafollicular			1.02	0.96–1.09	0.50	1.01	0.98–1.05	0.54	0.98	0.91–1.06	0.66
in interfollicular			0.98	0.93–1.03	0.39	0.98	0.95–1.01	0.25	1.01	0.96–1.07	0.68
CD8^+^ cells in intrafollicular			0.91	0.83–1.00	0.053	0.92	0.86–0.97	0.0038	0.95	0.86–1.05	0.30
in interfollicular			1.03	0.97–1.09	0.28	1.03	0.99–1.07	0.093	1.06	0.99–1.14	0.064
CD68^+^ cells in intrafollicular			0.85	0.72–0.99	0.045	0.95	0.90–1.02	0.14	0.91	0.80–1.03	0.13
in interfollicular			0.99	0.92–1.07	0.87	1.01	0.97–1.06	0.61	1.05	0.97–1.14	0.22

## Data Availability

The data that support the findings of the current study are available from the corresponding author, N.H., upon reasonable request.

## Supplementary Materials

The following supporting information can be downloaded at https://www.mdpi.com/article/10.3390/ijms252011057/s1.
