# Supplementary material for: High Ki-67 Expression Predicting a Risk Factor for the Progression of Disease within 24 Months and Microenvironment in Follicular Lymphoma
Source: Int J Mol Sci. 2024 Oct 15;25(20):11057. doi: 10.3390/ijms252011057 (PMC11507466; doi:10.3390/ijms252011057)
Supplement: Supplementary file 1 [file ijms-25-11057-s001.zip › ijms-3247332-supplementary.pdf]

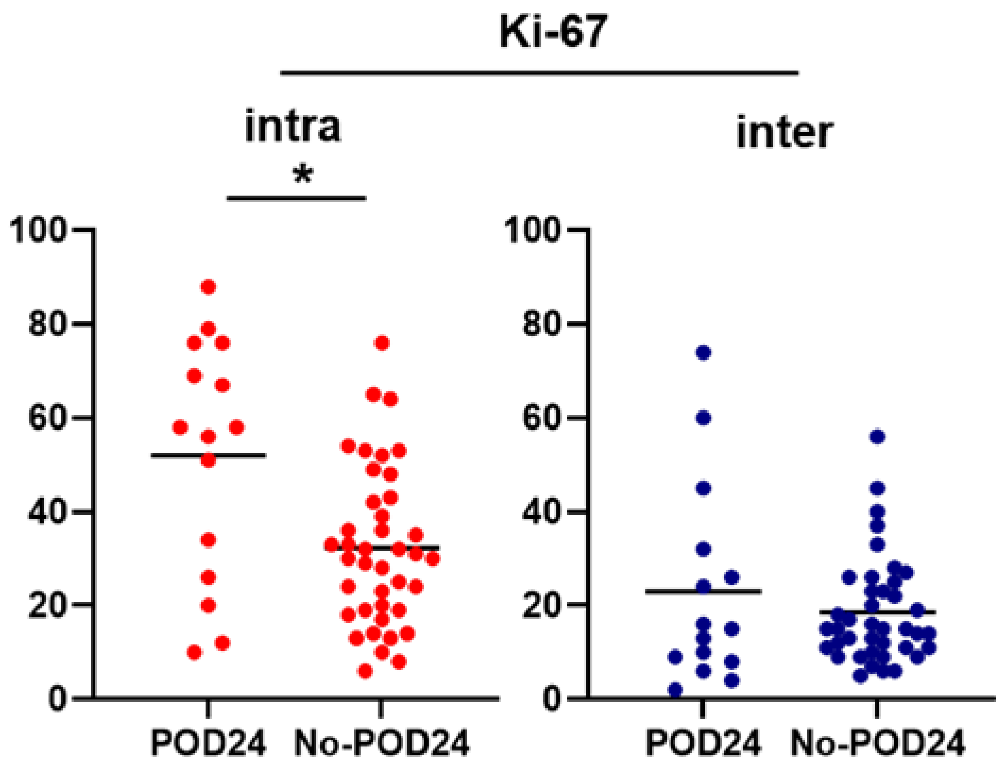

Figure S1. Comparison of Ki-67 positive cells between patients with FL with and without POD24 according to separately counted intrafollicular and interfollicular areas.
